# Nourishing futures: Assessing nutritional health among children under-five years of age belonging to a particularly vulnerable tribal community in Southern India

**DOI:** 10.1371/journal.pone.0348880

**Published:** 2026-05-11

**Authors:** Sneha Deepak Mallya, Biju Soman, Srikanth Gouspure, Pavan Kumar Thatoju, Dinesh M. Nayak, Ashwini Kumar, Harpreet Kaur, Ranjitha S. Shetty

**Affiliations:** 1 Department of Community Medicine, Kasturba Medical College, Manipal Academy of Higher Education, Manipal, India; 2 Department of Pediatrics, Melaka Manipal Medical College (Manipal Campus), Manipal Academy of Higher Education, Manipal, India; 3 Division of Epidemiology and Communicable Diseases, Indian Council of Medical Research, Department of Health Research, Ministry of Health and Family Welfare, New Delhi, India; West Bengal State University, INDIA

## Abstract

Malnutrition is a major public health concern among tribal communities, where children face higher risk due to socio-economic disadvantages, poor healthcare access, and inadequate diets. Despite government programs, it persists, necessitating a focused assessment of its determinants. This study aimed to evaluate the nutritional status and its determinants among Koraga tribal children. A community-based cross-sectional study was conducted among children under-five years of age belonging to the Koraga community, a particularly vulnerable tribal group (PVTG) in southern Karnataka, India. Anthropometric measurements were taken, and data on socio-demographic characteristics, maternal and child health, and environmental conditions were collected using a structured proforma. Data analysis was performed using SPSS version 16.0 with logistic regression identifying factors associated with malnutrition. Among the 180 children included in the study, the median [interquartile range (IQR)] age of the children was 36 months (22.25, 46.00), and 52.2% were female. Low birth weight was reported in 40.2% of children, and 9.9% were currently anaemic. The study found a high prevalence of malnutrition among children under-five years of age, with 81.1% being stunted, 78.3% wasted, and 80.6% underweight. Multivariable analysis identified access to safe drinking water, maternal factors, nutritional factors, and dietary factors as significant contributors to undernutrition in this vulnerable population. The findings highlight the urgent need for targeted nutrition and health interventions in this PVTG. Strengthening maternal care, child feeding practices, sanitation, and healthcare access is essential to reduce malnutrition. A multi-sectoral approach is essential to break the cycle of undernutrition and improve overall child health outcomes.

## Introduction

Malnutrition is a vicious cycle in both the general population and tribal communities, but it is often more severe among tribal populations due to greater socio-economic disadvantages, traditional practices, geographical isolation, limited health services, cultural beliefs, and lower literacy rates exacerbating the cycle. Malnutrition, as classified by the World Health Organization (WHO), includes both undernutrition (wasting, stunting, underweight, and micronutrient deficiencies) and overnutrition (overweight and obesity). Wasting (low weight for height/length) indicates acute malnutrition, while stunting (low height/length for age) reflects chronic undernutrition and underweight (low weight for age) can result from both, affecting growth and survival [1]. In 2022, 149 million children under-five years of age were stunted, 45 million were wasted, and 37 million were overweight or obese. Nearly half of deaths among children who are under-five years of age are linked to undernutrition, with lasting impacts in low- and middle-income countries [2,3]. Malnutrition, low birth weight (LBW), and diarrhea are major health problems among the tribal population of India. Many tribal individuals initially seek care from traditional healers and turn to public health facilities only when necessary. As a result, tribal children suffer from higher levels of malnutrition and increased rates of illness compared to their non-tribal counterparts [4–7].

The National Nutrition Monitoring Bureau (NNMB) 3rd Repeat Survey-2012 and Comprehensive National Nutrition Survey (CNNS) 2016–18 from India have shown a high burden of malnutrition among Scheduled Tribe (ST) children. According to the CNNS report, 41.5% of ST children under the age of five years were stunted, 21.9% were wasted, and 41.5% were underweight, indicating severe nutritional challenges [8]. Data from NNMB shows that ST children had the highest rates of underweight (54%), stunting (54%), and wasting (29%). These findings highlight severe health inequalities, caused by poverty, poor diet, lack of healthcare, and socio-cultural factors [9]. Malnutrition among ST children remains higher than in the general population. According to the National Family and Health Survey-5 (NFHS-5) of India, 40.9% of ST children are stunted, 23.2% are wasted, and 39.5% are underweight, compared to 35.5%, 19.3%, and 32.1% in the general population, respectively [10]. Malnutrition is also highly prevalent among tribal children under-five years of age in Karnataka, as indicated by stunting at 39.5%, wasting at 21.5%, and underweight at 35.8%. Additionally, 69.6% of children under-five years of age have anaemia. Further, the district of Udupi, where the present study was conducted, reported lower rates as compared to the state and national average with stunting at 23.1%, wasting at 17.6%, and underweight at 21.0% among children under-five years of age [11]. Of the 75 Particularly vulnerable tribal groups (PVTG) identified by the government of India, the two tribes namely Koraga and Jenukuruba from Karnataka, have poorer health status than other communities, due to poverty, poor sanitation, limited healthcare access, and their remote locations [12–14].

Tribal populations in India face multiple health challenges, including malnutrition, communicable and non-communicable diseases (NCDs), and mental health disorders. Traditional beliefs, substance abuse, and various socio-economic and cultural factors contribute to poor health outcomes, resulting in higher malnutrition rates and limited access to healthcare. Among tribal children, socioeconomic status, indigenous background, parental factors, child-related factors, poor hygiene, and child illnesses are key contributors to stunting, wasting, and underweight, increasing the risk of morbidity and mortality [15,16]. PVTGs have poorer health indicators compared to both the general and other tribal populations. Limited access to maternal and child healthcare, poor health-seeking behavior, and high rates of substance abuse contribute to their vulnerability to malnutrition, anemia, and infectious diseases [17]. The Koraga community, a PVTG in coastal Karnataka, remains socially marginalized with limited access to health care due to geographical isolation, social stigma, language barriers and socioeconomic challenges. Prior findings from Koraga community indicated that 42.3% of under 5 children were underweight, 30.8% were stunted and 15.4% were wasted [12,18]. Addressing these issues aligns with Sustainable Development Goal (SDG) 2 (Zero Hunger) which emphasizes improving nutrition and SDG 3, which aims to ensure healthy lives and well-being for all [19]. Therefore, this study aimed to assess the nutritional status and its determinants among children under-five years of age belonging to the Koraga community, a PVTG residing in the Udupi district.

## Materials and methods

The study was approved by the Ethics Committee of Kasturba Medical College and Kasturba Hospital (IEC:747/2021). The data was collected from 3/12/2021–31/10/2022. After explaining the details about the study in the local language Kannada, a written informed consent was obtained from the mothers of children under-five years of age. For mothers who were illiterate a thumb impression was taken in the presence of a witness along with their signature who could read, write and understand the study related information. Community engagement was facilitated through local leaders and health workers.

Additional information regarding the ethical, cultural, and scientific considerations specific to inclusivity in global research is included in the supporting information (S1 File).

This community-based cross-sectional study was conducted among the children under-five years of age belonging to Koraga tribe residing in Udupi district. The district of Udupi is located in Karnataka, a south Indian state. The Koraga population resides across seven blocks in Udupi District including Kundapura, Kaup, Karkala, Byndoor, Udupi, Hebri and Brahmavara. A complete enumeration sampling approach was adopted. All the tribal hamlets were visited, and eligible children were included. After complete enumeration of all eligible children in the study setting, a total of 180 children constituted for the final sample size of the study. The list of eligible houses of the Koraga community was obtained prior to initiation of the survey from Integrated Tribal Development Project (ITDP) Office, Udupi, an administrative body by Government of India to support the socio-economic development of scheduled tribes. Following this, door-to-door visits were conducted with the help of local community leaders and the purpose of the visit was explained to the mothers of children within the family. All the eligible children in a given household who were aged ≤ five years were recruited into the study. Socio-demographic details of the household were collected by administering a pre-tested, semi-structured questionnaire for the mothers of children with the assistance of a qualified nurse serving as a research assistant. Socio-economic status was assessed by using modified BG Prasad’s scale [20]. Details regarding prenatal, natal and post-natal history, breastfeeding, feeding practices, immunization, and any recent infections were collected. Details pertaining to utilization of nutritional supplementation services provided through Integrated Child Development Services (ICDS) scheme and rations obtained from ITDP were collected. The ICDS program, is a flagship initiative of the Government of India, that provides essential nutrition, healthcare, and early education to children under six years old, significantly improving their health and development outcomes through Anganwadi centers [21]. Accredited social health activist (ASHA) at gross root level supports Anganwadi centers by mobilizing pregnant, lactating women and infant/children for nutritional supplementation and related health services through regular home visits. The food packets provided by ITDP include essential items such as rice, pulses, oil, and other basic food supplies to ensure balanced nutrition. A detailed physical examination was carried out by the study nurse among all the recruited children to look for signs of nutritional deficiency, which included the presence of pallor, cheilosis/angular stomatitis (Vitamin B2 deficiency) and Bitot’s spots (Vitamin A deficiency). History of acute respiratory infections and acute diarrheal disorders, chronic ingestion of inedible substances (pica) and worm infestation were enquired about. Nutritional intake of mothers and children was assessed by using the 24 hours dietary recall method with respect to calories, protein, vitamin A and iron intake as per National Institute of Nutrition (NIN) guidelines [22]. Length and weight for age and weight for length were assessed for children aged 0–24 months; height and weight for age and weight for height were assessed for children aged 24–59 months using the WHO 2006 guidelines. Stunting, wasting and underweight are key anthropometric indicators of child malnutrition. Children with height for age, weight for height or weight for age, Z scores below −2 standard deviations are classified as stunted, wasted or underweight respectively [23]. The mothers’ height and weight were measured and categorized based on the body mass index (BMI) values as per WHO classification [24]. Screening for anemia was carried out through finger prick method using a handheld portable hemoglobinometer for both mothers and children (1–5 years) at the household level and was classified according to WHO guidelines [25]. Peripheral smears were done using finger prick method for those children who were found to be anemic and mothers who had moderate and severe anemia and the slides were tested at Department of Pathology of the medical college. Data was entered and analyzed using SPSS version 16.0. Results have been expressed as frequencies and proportions. Continuous variables are presented as mean or median with the standard deviation (SD) or interquartile range (IQR) respectively wherever applicable. Variables with a p-value ≤0.2 on univariate analysis were included for multivariable analysis. Multivariable logistic regression analysis was done to find out the association between stunting, wasting and underweight and various background characteristics, including maternal and child-related parameters. The strength of association has been expressed as unadjusted odd’s ratio (OR) and adjusted OR (AOR) with 95 per cent confidence interval (CI). A p-value <0.05 was considered statistically significant.

## Results

The study included a total of 180 households and a third of the households were from Kundapura block. The proportion of households included from other six blocks were as follows: Kaup (14.4%), Karkala (14.4%), Byndoor (14.4%), Udupi (11.2%), Hebri (8.3%), Brahmavara (5.6%). The background characteristics of the households included in the study are depicted in Table 1. Among the study participants, 91.1% possessed their own houses, though 133 (73.9%) lived in semi-pucca houses. Of the households surveyed, 168 (93.3%) reported having an electrical connection, and only 37.2% of the families used LPG as cooking fuel. Almost one-fourth (23.3%) of them did not have a habit of treating water before consuming, and 7.2% of the families did not have a household toilet. Further, more than two-thirds (69.4%) of the families possessed a below poverty line (BPL) ration card, and 171 (95.0%) reported having received ITDP food packets.

**Table 1:** Background characteristics of the surveyed households (n = 180).

Characteristics	Categories	n (%)
Type of family	Nuclear	48 (26.7)
	Joint	112 (62.2)
	Third generation	20 (11.1)
Family members	≤ 5 members	69(37.8)
	>6 members	112 (62.2)
Socioeconomic status	Upper middle (3766–7532)	9 (5.0)
	Middle (2260–3765)	41 (22.8)
	Lower middle (1130–2259)	72 (40.0)
	Lower (≤1129)	58 (32.2)
Possession of Ration Card	Yes	170 (94.4)
	No	10 (5.6)
Type of house	Kutcha House	34 (18.9)
	Semi-Pucca house	133 (73.9)
	Pucca house	13 (7.2)
Type of cooking Fuel	Wood stove	113 (62.8)
	Liquid Petroleum Gas	67 (37.2)
Source of drinking water	Open well	96 (53.3)
	Bore well	84 (46.7)
Drinking water treatment before use	Yes	138 (76.7)
	No	42 (23.3)
Presence of a household toilet	Yes	167 (92.8)
	No	13 (7.2)

Table 2 depicts reproductive characteristics, dietary intake and nutritional profile of surveyed mothers. The majority (74.4%) of mothers had completed 5–10 years of schooling. The mean (SD) age at marriage and first conception was 22.72(4.66) years and 24.07(4.83) years, respectively. Among the study participants, 18(10.0%) had conceived more than three times.

**Table 2:** Reproductive characteristics, dietary intake and nutritional profile of the surveyed mothers (n = 178).

Characteristics	Categories	n (%)
Educational status	Illiterate up to 4 years of schooling	25 (13.9)
	5-10 years of schooling	134 (74.4)
	>10 years of schooling	21(11.7)
Age at marriage (in years)	15-17	19(10.6)
	≥18-25	161(89.4)
Age at first conception (in years)	<18	5(2.8)
	18-30	153(85.0)
	>30	22(12.2)
Number of times conceived	Once	90 (50.0)
	Twice	72 (40.0)
	Thrice	12 (6.7)
	>than 3 times	6 (3.3)
Type of delivery	Normal	99 (55.0)
	Cesarean section	81 (45.0)
Calorie intake of mother	Inadequate	178 (100.0)
Protein intake of mother	Adequate	8 (4.5)
	Inadequate	170 (95.5)
Calcium intake of mother	Inadequate	178 (100.0)
Iron intake of mother	Adequate	3 (1.7)
	Inadequate	175 (98.3)
Retinol intake of mother	Adequate	20 (11.2)
	Inadequate	158 (88.8)
BMI of mother	Under weight	81 (45.0)
	Normal weight	77 (42.8)
	Overweight &Obesity	22 (12.2)
Hemoglobin level of mother	Normal	100 (56.1)
	Mild anaemia	41 (23.1)
	Moderate anaemia	14(7.9)
	Severe anaemia	23 (12.9)

Note: One mother had twins and another mother had two children under five years, resulting in a total of 178 mothers.

While querying about dietary intake, it was found that the calorie and calcium intake were inadequate among all mothers. On anthropometric assessment, almost half (45.0%) of the mothers were found to be underweight, and 43.9% were found to have anaemia on hemoglobin estimation. Peripheral smear examination was conducted among 30 mothers diagnosed with moderate to severe anaemia. Of these, two were found to have microcytic anaemia, while the remaining 28 exhibited normocytic anaemia. Peripheral smear could not be performed on seven mothers.

Mothers of children under-five years of age were asked about their source of information regarding the services provided by the Anganwadi (a center under ICDS for delivering the services related to childcare and nutrition). The responses included their mothers (148, 82.3%), health workers (25,13.9%) and other relatives (7,3.9%). The participants were aware of the following services: nutritious food (123, 68.3%), non-formal pre-school education (49, 27.2%) and vaccination (5, 2.8%). About three mothers (1.7%) were not aware of any services provided by the Anganwadi.

Characteristics of children, including details on breastfeeding, current nutritional intake, anthropometric parameters and morbidity are depicted in Table 3. The median (IQR) age of the children was 36.00 (22.25, 46.00) months, 52.2% were female and 40.2% of children were born with low birth weight. Of the surveyed children, 145(80.6%) were underweight, 146 (81.1%) were stunted and 141(78.3%) were wasted. Hemoglobin was estimated among 161 children and was found to be normal among 145 (90.1%). The mean (SD) hemoglobin level was 10.93(3.37) gm/dl. Peripheral smear examination was performed on 13 out of 16 children diagnosed with anaemia and three children exhibited microcytic anaemia, while the remaining ten showed normocytic anaemia. Calorie, calcium and iron intake were inadequate among the majority of children.

**Table 3:** Anthropometry, dietary and morbidity profile of children under-five years of age (n = 180).

Characteristics	Categories	n (%)
Age (in months)	0-12	11 (6.1)
	13-24	47 (26.1)
	25-60	122 (67.8)
Sex	Male	86 (47.8)
	Female	94 (52.2)
Hospitalization during neonatal period	Yes	21 (11.7)
	No	159(88.3)
Birth weight	Very low birth weight	10 (5.6)
	Low birth weight	62 (34.6)
	Normal weight	107 (59.8)
Weight for age	Severe underweight	21 (11.7)
	Moderate underweight	75 (41.7)
	Mild underweight	49 (27.2)
	Normal weight	35 (19.4)
Height for age	Severe Stunting	23 (12.8)
	Moderate Stunting	35 (19.4)
	Mild Stunting	88 (48.9)
	normal status	34 (18.9)
Weight for height	Severe wasting	46 (25.6)
	Moderate wasting	34 (18.9)
	Mild wasting	61 (33.9)
	Normal status	39 (21.7)
Hemoglobin level	Normal	145 (90.1)
	Mild anaemia	1(0.6)
	Moderate anaemia	10 (6.2)
	Severe anaemia	5 (3.1)
Type of anaemia	Normocytic anemia	10 (5.5)
	Microcytic anemia	3 (1.7)
Immunization status	Fully immunized	176 (97.8)
	Partially immunized	4 (2.2)
History of Measles in the last six months	Yes	9 (5.0)
	No	171 (95.0)
History of Acute Respiratory Infection/Acute Diarrheal Disease in the last six months	Yes	11(6.1)
	No	169(93.9)
Worm infestation in last six months	Yes	15 (8.3)
	No	165 (91.7)
History of Ingestion of clay/soil	Yes	18 (10.0)
	No	162 (90.0)
Hospitalization of child in the last year for acute illness	Yes	36 (20.0)
	No	144 (80.0)
Calorie intake	Adequate	34 (18.9)
	Inadequate	146 (81.1)
Protein intake	Adequate	119 (66.1)
	Inadequate	61 (33.9)
Calcium intake	Adequate	7 (3.9)
	Inadequate	173 (96.1)
Iron intake	Adequate	37 (20.6)
	Inadequate	143 (79.4)
Retinol intake	Adequate	71 (39.4)
	Inadequate	109(60.6)
Breast feeding initiation	Immediately after birth	107 (59.4)
	Half an hour-4 hours	68 (37.7)
	Beyond 4 hours	5 (2.7)
Pre-lacteal feeding to the infant	Yes	21 (11.7)
	No	159 (88.3)
Duration of breastfeeding to child (in months)	0 - 6	10 (5.6)
	7 −12	12 (6.7)
	13 −24	60 (33.3)
	> 24	98 (54.4)
Whether mother breastfeeds when she is sick	Yes	134 (74.4)
	No	46 (25.6)
Whether mother breastfeeds when the child is sick	Yes	137 (76.1)
	No	43 (23.9)
Whether washes hands before feeding the child	Yes	118 (65.6)
	No	10 (5.6)
	Sometimes	52 (28.8)
Washes fruits and vegetables	Yes	176 (97.7)
	No	4 (2.2)
Freshly prepares meals	Yes	122 (67.8)
	No	58 (32.2)
Supplementary nutrition provided by ICDS is utilized by child	Yes	153 (85.0)
	No	23 (12.8)
	Not sure	4 (2.2)
Whether house is visited by accredited social health activist (ASHA)/ Community health officer (CHO)^a^	Yes	165(91.7)
	No	15(8.3)
Presence of nearby health facility	Yes	175(97.2)
	No	5(2.8)

Note: Nine children were not tested for anaemia since they were below one year, 10 children refused haemoglobin test.

(CHO)^a^ – grassroot level workers who are responsible for house visit at the subcenter or primary health center level.

Table 4 depicts univariate and multivariable logistic regression analysis between stunting and sociodemographic, maternal and child characteristics.

**Table 4:** Association between stunting and sociodemographic, maternal and child characteristics (n = 180).

Characteristics	Category	Stunting		COR (95%CI)	p value	AOR (95%CI)	p value
		Present (n = 146) n(%)	Absent (n = 34) n(%)				
**Sociodemographic characteristics**							
Type of family	Nuclear	44(91.7)	4(8.3)	1.22 (0.02-7.27)	0.82	1.52 (0.55-42.13)	0.803
	Joint	84(75.0)	28(25.0)	0.33 (0.07-1.52	0.15	0.17 (0.01-1.809)	0.14
	Third generation	18(90.0)	2(10.0)	1		1	
Number of Family members	≤5 members	59(86.8)	9(13.2)	1		1	
	>6 members	87(77.7)	25(22.3)	0.053 (0.23-1.21)	0.13	0.28 (0.04-1.98)	0.206
Socioeconomic status	Middle	102(83.6)	20(16.4)	1		1	
	Lower	44(75.9)	14(24.1)	0.61 (0.28-1.33)	0.21	0.69 (0.17-2.69)	0.59
Type of ration card	APL	10(90.9)	1(9.1)	1		–	
	BPL/ Antyodaya/No ration card	136(80.5)	33(19.5)	0.41 (0.05-3.33)	0.406	–	
Type of house	Kutcha House	25(73.5)	9(26.5)	0.23 (0.02-2.04)	0.18	0.05 (0.003-1.209)	0.66
	Semi-Pucca house	109(82.0)	24(18.0)	0.37 (0.04-3.05)	0.36	0.33 (0.02-5.54)	0.44
	Pucca house	12(92.3)	1(7.7)	1		1	
Type of cooking fuel	Wood stove	94(83.2)	19(16.8)	1.42 (0.66-3.04)	0.35	–	
	LPG stove	52(77.6)	15(22.4)	1		–	
Source of drinking water	Open well	83(86.5)	13(13.5)	2.12 (0.99-4.57)	0.05	4.02 (1.08-14.89)	0.03*
	Bore well	63(75.0)	21(25.0)	1		1	
Drinking water treatment before use	Yes	107(77.5)	31(22.5)	1		1	
	No	39(92.9)	3(7.1)	3.76 (1.08-13.02	0.03	7.26 (0.80-65.73)	0.07
Presence of household toilet	Yes	135(80.8)	32(19.2)	1		–	
	No	11(84.6)	2(15.4)	1.30 (0.27-6.17)	0.73	–	
**Maternal characteristics**							
Educational status of mother	Illiterate or upto 4 years of schooling	20(80.0)	5(20.0)	1.25 (0.307-5.08)	0.75	–	
	5-10 years of schooling	110(82.1)	24(17.9)	1.43 (0.47-4.29)	0.52	–	
	>10 years of schooling	16(76.2)	5(23.8)	1		–	
Education of spouse	Illiterate or upto 4 years of schooling	51(83.6)	10(16.4)	1.02 (0.28-3.63)	0.97	–	
	5-10 years of schooling	75(78.9)	20(21.1)	0.75 (0.23-2.44)	0.63	–	
	>10 years of schooling	20(83.3)	4(16.7)	1		–	
Age at marriage (in years)	<18	14(73.7)	5(26.3)	0.61 (0.205-1.84)	0.38	–	
	≥18	132(82.0)	29(18.0)	1		–	
Age at first conception (in years)	≤30	129(81.6)	29(18.4)	1.308 (0.446-3.83)	0.62	–	
	>30	17(77.3)	5(22.7)	1		–	
Number of times conceived	Once	69(76.7)	21(23.3)	1		1	
	≥ Two	77(85.6)	13(14.4)	1.803 (0.84-3.87)	0.13	4.47 (1.03-19.31)	0.04*
Number of ANC check ups	<Four visits	9(90.0)	1(10.0)	2.16 (0.26-17.71)	0.47	–	
	≥Four visits	137(80.6)	33(19.4)	1		–	
Type of delivery	Normal	77(77.8)	22(22.2)	1		1	
	Cesarean section	69(85.2)	12(14.8)	1.64 (0.75-3.56)	0.209	2.83 (0.76-10.58)	0.12
Calorie intake among women	Inadequate	34(19.1)	144(80.9)	–		–	
Protein intake among women	Adequate	6(75.0)	2(25.0)	1		–	
	Inadequate	138(81.2)	32(18.8)	1.43 (0.27-7.45)	0.66	–	
Calcium intake among women	Inadequate	144(80.9)	34(19.1)	–		–	
Iron intake among women	Adequate	2(66.7)	1(33.3)	1		–	
	Inadequate	142(81.1)	33(18.9)	2.15 (0.18-24.44)	0.53	–	
Retinol intake among women	Adequate	12(60.0)	8(40.0)	1			
	Inadequate	132(83.5)	26(16.5)	3.38 (1.26-9.09)	0.016	4.81 (0.706-32.807)	0.108
BMI of mother	Under weight	65(80.2)	16(19.8)	1.15 (0.53-2.48)	0.71	0.22 (0.04-1.03)	0.05
	Normal weight	60(77.9)	17(22.1)	1		1	
	Overweight & Obesity	21(95.5)	1(4.5)	5.95 (0.74-47.48)	0.09	28.50 (0.32-2535.66)	0.14
Hemoglobin level of mother	Normal	81(81.0)	19(19.0)	1		–	
	Mild anaemia	33(80.5)	8(19.5)	1.56 (0.43-5.80)	0.504	–	
	Moderate anaemia	10(71.4)	4(28.6)	0.58 (0.16-2.07)	0.407	–	
	Severe anaemia	20(87.0)	3(13.0)	0.96 (0.38-2.42)	0.94	–	
**Characteristics of children**							
Age (in months)	Below 24 months	50(86.2)	8(13.8)	1		1	
	25-60 months	96(78.7)	26(21.3)	0.59 (0.24-1.40)	0.23	0.34 (0.08-1.45)	0.14
Sex	Male	71(82.6)	15(17.4)	1		–	
	Female	75(79.8)	19(20.2)	0.83 (0.39-1.76)	0.63	–	
Hospital admission in neonatal period	Yes	18(85.7)	3(14.3)	1.45 (0.403-5.24)	0.56	–	
	No	128(80.5)	31(19.5)	1		–	
Birth weight	Low birth weight	63(87.5)	9(12.5)	2.13 (0.93-4.89)	0.07	6.69 (1.68-26.59)	0.007*
	Normal weight	82(76.6)	25(23.4)	1		1	
Immunization status	Fully immunized	145(82.4)	31(17.6)	1		1	
	Partially immunized	1(25.0)	3(75.0)	0.07 (0.007-0.708)	0.024	0.11 (0.003-4.38)	0.24
Positive history of Measles in the last six months	Yes	6(66.7)	3(33.3)	0.44 (0.105-1.86)	0.26	–	
	No	140(81.9)	31(18.1)	1		–	
History of ARI/ADD in last six months	Yes	8(72.7)	3(27.3)	0.59 (0.15-2.38)	0.46	–	
	No	138(81.7)	31(18.3)	1		–	
Worm infestation in last six months	Yes	9(60.0)	6(40.0)	0.30 (0.10-0.93)	0.03	0.05 (0.006-0.47)	0.008*
	No	137(83.0)	28(17.0)	1		1	
History of Ingestion of clay/soil	Yes	14(77.8)	4(22.2)	0.79 (0.24-2.58)	0.704	–	
	No	132(81.)	30(18.5)	1		–	
Hospitalization of child in last one year for acute illness	Yes	27(75.0)	9(25.0)	0.63 (0.26-1.503)	0.29	–	
	No	119(82.6)	25(17.4)	1		–	
Calorie intake	Adequate	24(70.6)	10(29.4)	1		1	
	Inadequate	122(83.6)	24(16.4)	2.11 (0.89-4.99)	0.08	0.49 (0.08-2.75)	0.42
Protein intake	Adequate	94(79.0)	25(21.0)	1		–	
	Inadequate	52(85.2)	52(85.2)	1.52 (0.66-3.53)	0.31	–	
Calcium intake	Adequate	5(71.4)	2(28.6)	1		–	
	Inadequate	141(81.5)	32(18.5)	1.76(0.32-9.49)	0.51	–	
Iron intake	Adequate	25(67.6)	12(32.4)	1		1	
	Inadequate	121(84.6)	22(15.4)	2.64 (1.15-6.02)	0.02	2.11 (0.45-9.82)	0.34
Retinol intake	Adequate	50(70.4)	21(29.6)	1		1	
	Inadequate	96(88.1)	13(11.9)	3.10 (1.43-6.709)	0.004	6.31 (1.54-25.83)	0.01*
Breast feeding initiation	Immediately after birth	87(81.3)	20(18.7)	1		–	
	Half an hour-4 hours	55(80.9)	13(19.1)	0.97 (0.44-2.11)	0.94	–	
	Beyond 4hrs	4(80.0)	1(20.0)	0.92 (0.09-8.67)	0.94	–	
Pre lacteal feeding to infant	Yes	14(66.7)	7(33.3)	0.409 (0.15-1.109)	0.07	2.98 (0.58-15.209)	0.18
	No	132(83.0)	27(17.0)	1		1	
Duration of breast feeding to child (months)	Upto 6	6(60.0)	4(40.0)	0.36 (0.09-1.407)	0.14	0.04 (0.005-0.39)	0.005*
	7 −12	8(66.7)	4(33.3)	0.48 (0.13-1.76)	0.27	0.11 (0.009-1.38)	0.08
	13 −24	53(88.3)	7(11.7)	1.82 (0.71-4.63)	0.208	1.06 (0.19-5.88)	0.94
	> 24	79(80.6)	19(19.4)	1		1	
Whether mother breastfeeds when she is sick	Yes	106(79.1)	28(20.9)	1		1	
	No	40(87.0)	6(13.0)	1.76 (0.67-4.57)	0.24	1.78 (0.34-9.12)	0.48
Whether mother breastfeeds when child is sick	Yes	110(80.3)	27(19.7)	1		–	
	No	36(83.7)	7(16.3)	1.26 (0.507-3.14)	0.61	–	
Whether washes hand before feeding the child	Yes	94(79.7)	24(20.3)	1		–	
	No	9(90.0)	1(10.0)	2.29 (0.27-19.03)	0.44	–	
	Sometimes	43(82.7)	9(17.3)	1.22 (0.52-2.84)	0.64	–	
Washes fruits and vegetables	Yes	143(81.2)	33(18.8)	1		–	
	No	3(75.0)	1(25.0)	0.69 (0.07-6.86)	0.75	–	
Freshly prepares meals	Yes	98(80.3)	24(19.7)	1		–	
	No	48(82.8)	10(17.2)	1.17 (0.52-2.65)	0.69	–	
Supplementary nutrition utilized by child	Yes	123(80.4)	30(19.6)	1		–	
	No	20(87.0)	3(13.0)	1.62 (0.45-5.83)	0.45	–	
	Not sure	3(75.0)	1(25.0)	0.73 (0.07-7.28)	0.79	–	
Whether house is visited by accredited social health activist (ASHA)/ Community health officer (CHO)^a^	Yes	136(82.4)	29(17.6)	1		1	
	No	10(66.7)	5(33.3)	0.42 (0.13-1.34)	0.14	0.08 (0.01-0.77)	0.02*
Presence of nearby health facility	Yes	143(81.7)	32(18.3)	1		1	
	No	3(60.0)	2(40.0)	0.33 (0.05-2.09)	0.24	0.11 (0.000108-124.92)	0.11

Note: Nine children were not tested for anaemia since they were below one year, 10 children refused haemoglobin test.

(CHO)^a^ – grassroot level workers who are responsible for house visit at the subcentre or primary health centre level.

*statistically significant.

Univariate analysis showed an association of stunting with source of drinking water, treatment of drinking water before use, maternal retinol intake, low birth weight, worm infestation, retinol and iron intake of the children. Further, on multivariable analysis, the source of drinking water (AOR: 4.02; CI: 1.08–14.89; p = 0.03), a higher number of maternal pregnancies (AOR: 4.47; CI: 1.03–19.31; p = 0.04), low birth weight (AOR: 6.69; CI: 1.68–26.59; p = 0.007) and retinol inadequacy (AOR: 6.31; CI: 1.54–25.83; p = 0.01) were positively associated with stunting. However, lack of exclusive breastfeeding for the first six months, inadequate home visits by ASHA/CHO and worm infestation in the children showed a negative association with stunting. These negative associations may be influenced by the high prevalence of stunting within the study population or the limited sample size in certain categories of these variables.

Similarly, for wasting among children under-five years of age, univariate and multivariable analyses showed significant associations with age of the child and poor dietary protein intake as depicted in Table 5. The present study results indicate that children aged 25−60 months had a higher likelihood of wasting (AOR: 5.05; CI: 2.13–11.99; p < 0.001) while inadequate protein intake was associated with a lower likelihood of wasting (AOR: 0.27; CI: 0.11–0.69; p = 0.006). This may be attributed to the fact that most of the children were wasted.

**Table 5:** Association between wasting and sociodemographic, maternal and child characteristics (n = 180).

Characteristic	Category	Wasting		COR with 95%CI	p value	AOR with 95%CI	p value
		Present (n = 141) n (%)	Absent (n = 39) n (%)				
**Sociodemographic characteristics**							
Type of family	Nuclear	39(81.2)	9(18.8)	1			
	Joint	86(76.8)	26(23.2)	0.76 (0.32-1.78)	0.53		
	Third generation	16(80.0)	4(20.0)	0.92 (0.24-3.43)	0.905		
Number of Family members	≤5 members	54(79.4)	14(20.6)	1			
	>6 members	87(77.7)	25(22.3)	0.902 (0.43-1.88)	0.78		
Socioeconomic status	Middle	94(77.0)	28(23.0)	1			
	Lower	47(81.0)	11(19.0)	1.27 (0.58-2.77)	0.54		
Type of ration card	APL	7(63.6)	4(36.4)	1		1	
	BPL/ Antyodaya/No ration card	134(79.3)	35(20.7)	2.18 (0.606-7.89)	**0.23**	4.47 (0.82-24.15)	0.08
Type of house	Kutcha House	24(70.6)	10(29.4)	0.43 (0.08-2.33)	0.33		
	Semi-Pucca house	106(79.7)	27(20.3)	0.71 (0.14-3.41)	0.67		
	Pucca house	11(84.6)	2(15.4)	1			
Type of cooking fuel	Wood stove	89(78.8)	24(21.2)	1.07 (0.51-2.22)	0.85		
	LPG stove	52(77.6)	15(22.4)	1			
Source of drinking water	Open well	77(80.2)	19(19.8)	1.26 (0.62-2.57)	0.51		
	Bore well	64(76.2)	20(23.8)	1			
Drinking water treatment before use	Yes	104(75.4)	34(24.6)	1		1	
	No	37(88.1)	5(11.9)	2.41 (0.88-6.64)	0.08	2.37 (0.62-9.01)	0.204
Presence of household toilet	Yes	131(78.4)	36(21.6)	1			
	No	10(76.9)	3(23.1)	0.91 (0.23-3.505)	0.89		
**Maternal characteristics**							
Educational status of mother	Illiterate or upto 4 years of schooling	18(72.0)	7(28.0)	1.02 (0.28-3.72)	0.96		
	5-10 years of schooling	108(80.6)	26(19.4)	1.66 (0.58-4.69)	0.33		
	>10 years of schooling	15(71.4)	6(28.6)	1			
Education of spouse	Illiterate or upto 4 years of schooling	48(78.7)	13(21.3)	1.23 (0.406-3.73)	0.71		
	5-10 years of schooling	75(78.9)	20(21.1)	1.25 (0.43-3.56)	0.67		
	>10 years of schooling	18(75.0)	6(25.0)	1			
Age at marriage (in years)	<18	14(73.7)	5(26.3)	0.75(0.25-2.22)	0.604		
	≥18	127(78.9)	34(21.1)	1			
Age at first conception (in years)	≤30	122(77.2)	36(22.8)	0.53(0.15-1.91)	0.33		
	>30	19(86.4)	3(13.6)	1			
Number of times conceived	Once	70(77.8)	20(22.2)	1			
	≥ Two	71(78.9)	19921.1)	1.06 (0.52-2.17)	0.85		
Number of ANC check ups	<Four times	8(80.0)	2(20.0)	1.11 (0.22-5.46)	0.89		
	≥Four times	133(78.2)	37(21.8)	1			
Place of delivery	Hospital	137(78.3)	38(21.7)	1			
	Home	4(80.0)	1(20.0)	1.109 (0.12-10.22)	0.92		
Type of delivery	Normal	86(86.9)	13(13.1)	1			
	Cesarean section	73(90.1)	8(9.9)	1.07 (0.52-2.19)	0.84		
Calorie intake among women	Inadequate	139(78.1)	39(21.9)	–			
Protein intake among women	Adequate	7(87.5)	1(12.5)	1			
	Inadequate	132(77.6)	38(22.4)	0.49 (0.05-4.16)	0.51		
Calcium intake among women	Inadequate	139(78.1)	39(21.9)	–			
Iron intake among women	Adequate	3(100.0)	0(0)	–			
	Inadequate	136(77.7)	39(22.3)	–			
Retinol intake among women	Adequate	15(75.0)	5(25.0)	1			
	Inadequate	124(78.5)	34(21.5)	1.21 (0.41-3.58)	0.72		
BMI of mother	Under weight	67(82.7)	14(17.3)	1.46 (0.66-3.18)	0.34		
	Normal weight	59(76.6)	18(23.4)	1			
	Overweight & Obesity	15(68.2)	7(31.8)	0.65 (0.23-1.85)	0.42		
Hemoglobin level of mother	Normal	74(74.0)	26(26.0)	1		1	
	Mild anaemia	31(75.6)	10(24.4)	1.08 (0.47-2.52)	0.84	0.78 (0.28-2.12)	0.
	Moderate anaemia	13(92.9)	1(7.1)	4.56 (0.56-36.65)	0.15	9.43 (0.78-113.65)	0.07
	Severe anaemia	21(91.3)	2(8.7)	3.68 (0.809-16.82)	0.092	4.27 (0.71-25.51)	0.11
**Characteristics of children**							
Age (in months)	Below 24 months	35(60.3)	23(39.7)	1		1	
	25-60 months	106(86.9)	16(13.1)	4.35 (2.06-9.15)	<0.001	5.05 (2.13-11.99)	<0.001*
Sex	Male	68(79.1)	18(20.9)	1			
	Female	73(77.7)	21(22.3)	0.92 (0.45-1.87)	0.81		
Hospital admission in neonatal period	Yes	14(66.7)	7(33.3)	0.504 (0.18-1.35)	**0.17**	0.506 (0.15-1.66)	0.26
	No	127(79.9)	32(20.1)	1		1	
Birth weight	Low birth weight	58(80.6)	14(19.4)	1.26 (0.06-2.63)	0.53		
	Normal weight	82(76.6)	25(23.4)	1			
Immunization status	Fully immunized	137(77.8)	39(22.2)	–			
	Partially immunized	4(100.0)	0(0)	–			
Positive history of Measles in last six months	Yes	8(88.9)	1(11.1)	2.28 (0.27-18.85)	0.44		
	No	133(77.8)	38(22.2)	1			
History of ARI/ADD in last six months	Yes	6(54.5)	5(45.5)	0.302 (0.08-1.05)	0.06	0.22 (0.04-1.17)	0.07
	No	135(79.9)	34(20.1)	1		1	
Worm infestation in last six months	Yes	14(93.3)	1(6.7)	4.18 (0.53-32.89)	0.17	2.98 (0.28-30.84)	0.36
	No	127(77.0)	38(23.0)	1		1	
History of Ingestion of clay/soil	Yes	15(83.3)	3(16.7)	1.42 (0.39-5.209)	0.58		
	No	126(77.8)	36(22.2)	1			
Hospitalization of child in last one year for acute illness	Yes	25(64.9)	11(30.6)	0.54 (0.24-1.24)	0.15	0.83 (0.28-2.39)	0.73
	No	116(80.6)	28(19.4)	1		1	
Calorie intake	Adequate	27(79.4)	7(20.6)	1			
	Inadequate	114(78.1)	32(21.9)	0.92(0.36-2.31)	0.86		
Protein intake	Adequate	99(83.2)	20(16.8)	1			
	Inadequate	42(68.9)	19(31.1)	0.44 (0.21-0.92)	0.02	0.27 (0.11-0.69)	0.006*
Calcium intake	Adequate	6(85.7)	1(14.3)	1		1	
	Inadequate	135(78.0)	38(22.0)	0.59 (0.06-5.07)	0.63		
Iron intake	Adequate	27(73.0)	10(27.0)	1			
	Inadequate	114(79.7)	29(20.3)	1.45 (0.63-3.34)	0.37		
Retinol intake	Adequate	54(76.1)	17(23.9)	1			
	Inadequate	87(79.8)	22(20.2)	1.24 (0.607-2.55)	0.55		
Breast feeding initiation	Immediately after birth	82(76.6)	25(23.4)	1			
	Half an hour-4 hours	56(82.4)	12(17.6)	1.42 (0.66-3.06)	0.36		
	Beyond 4hrs	3(60.0)	2(40.0)	0.45 (0.07-2.89)	0.406		
Pre lacteal feeding to infant	Yes	17(81.0)	4(19.0)	1.200 (0.37-3.79)	0.75		
	No	124(78.0)	35(22.0)	1			
Duration of breast feeding to child (months)	Upto 6 months	8(80.0)	2(20.0)	0.96 (0.18-4.902)	0.96		
	7 −12 months	9(75.0)	3(25.0)	0.72(0.17-2.92)	0.64		
	13 −24 months	54(90.0)	6(10.0)	0.72 (0.33-1.55)	0.406		
	> 24 months	88(89.8)	10(10.2)	1			
Whether mother breastfeeds when she is sick	Yes	119(88.9)	15(11.2)	1			
	No	40(87.0)	6(13.0)	0.99 (0.44-2.24)	0.98		
Whether mother breastfeeds when child is sick	Yes	121(88.3)	16(11.7)	1.56 (0.63-3.86)	0.32		
	No	38(88.4)	5(11.6)	1			
Whether washes hand before feeding the child	Yes	89(75.4)	29(24.6)	1		1	
	No	8(80.0)	2(20.0)	1.303 (0.26-6.48)	0.74	0.97 (0.13-6.92)	
	Sometimes	44(84.6)	8(15.4)	1.79 (0.75-4.24)	0.18	0.98 (0.32-3.006)	0.98
Washes fruits and vegetables	Yes	138(78.4)	38(21.6)	1			
	No	3(75.0)	1(25.0)	0.82 (0.08-8.17)	0.87		
Freshly prepares meals	Yes	93(76.2)	29(23.8)	1			
	No	48(82.8)	10(17.2)	1.49 (0.67-3.32)	0.32		
Supplementary nutrition utilized by child	Yes	119(77.8)	34(22.2)	1			
	No	19(82.6)	4(17.4)	1.35 (0.43-4.25)	0.601		
	Not sure	3(75.0)	1(25.0)	0.85 (0.08-8.507	0.89		
Whether house is visited by accredited social health activist (ASHA)/ Community health officer (CHO)^a^	Yes	128(77.6)	37(22.4)	1			
	No	13(86.7)	2(13.3)	1.87 (0.406-8.703)	0.42		
Presence of nearby health facility	Yes	137(78.3)	38(21.7)	1			
	No	4(80.0)	1(20.0)	1.109 (0.12-10.22)	0.92		

Note: Nine children were not tested for anaemia since they were below one year, 10 children refused Hemoglobin test.

(CHO)a – grassroot level workers who are responsible for house visit at the subcenter or primary health center level, *statistically significant.

As shown in Table 6, age ≤ 30 years at conception was four times more likely to be associated with underweight, however, this was not statistically significant (AOR: 4.47; 0.98–20.26; p = 0.05). Due to the very high proportion of underweight children in the study population, not having a household toilet and not preparing fresh meals showed a negative association with underweight.

**Table 6:** Association between sociodemographic, maternal and child characteristics and underweight n = 180.

Characteristic	Category	Underweight		COR with 95%CI	p value	AOR with 95%CI	p value
		Present (n = 159) n(%)	Absent (n = 21) n(%)				
**Sociodemographic characteristics**							
Type of family	Nuclear	40(83.3)	8(16.7)	0.26 (0.03-2.25)	0.22	0.109 (0.006-1.94)	0.13
	Joint	100(89.3)	12(10.7)	0.43 (0.05-3.57)	0.44	0.15(0.01-2.29)	0.17
	Third generation	19(95.0)	1(5.0)	1		1	
Number of Family members	≤5 members	57(83.8)	11(16.2)	1		1	
	>6 members	102(91.1)	10(8.9)	1.96 (0.78-4.91)	0.14	1.49 (0.37-6.03)	0.57
Socioeconomic status	Middle	105(86.1)	17(13.9)	1		1	
	Lower	54(93.1)	4(6.9)	2.18 (0.701-6.81)	0.17	2.83 (0.66-12.12)	0.16
Type of ration card	APL	8(72.7)	3(27.3)	1		1	
	BPL/ Antyodaya/No ration card	151(89.3)	18(10.7)	3.14 (0.76-12.93)	0.11	1.32 (0.19-9.13)	0.77
Type of house	Kutcha House	31(91.2)	3(8.8)	0.86(0.08-9.11)	0.901		
	Semi-Pucca house	116(87.2)	17(12.8)	0.56 (0.06-4.65)	0.56		
	Pucca house	12(92.3)	1(7.7)	1			
Type of cooking fuel	Wood stove	98(86.7)	15(13.3)	0.64 (0.23-1.74)	0.38		
	LPG stove	61(91.0)	6(9.0)	1			
Source of drinking water	Open well	86(89.6)	10(10.4)	1.29 (0.52-3.22)	0.57		
	Bore well	73(86.9)	11(13.1)	1			
Drinking water treatment before use	Yes	120(87.0)	18(13.0)	1			
	No	39(92.9)	3(7.1)	1.95 (0.54-6.97)	0.304		
Presence of household toilet	Yes	150(89.8)	17(10.2)	1		1	
	No	9(69.2)	4(30.8)	0.25 (0.07-0.91)	0.03	0.08 (0.01-0.48)	0.006*
**Maternal characteristics**							
Educational status of mother	Illiterate or upto 4 years of schooling	21(84.0)	4(16.0)	0.87 (0.17-4.43)	0.87		
	5-10 years of schooling	120(89.6)	14(10.4)	1.42 (0.37-5.46)	0.602		
	>10 years of schooling	18(85.7)	3(14.3)	1			
Education of spouse	Illiterate or upto 4 years of schooling	53(86.9)	8(13.1)	0.28 (0.03-2.43)	0.25		
	5-10 years of schooling	83(87.4)	12(12.6)	0.301 (0.03-2.43)	0.26		
	>10 years of schooling	23(95.8)	1(4.2)	1			
Age at marriage (in years)	<18	16(84.2)	3(15.8)	0.67 (0.17-2.53)	0.55		
	≥18	143(88.8)	18(11.2)	1			
Age at first conception (in years)	≤30	142(89.9)	16(10.1)	2.61 (0.84-8.02)	0.09	4.47 (0.98-20.26)	0.05
	>30	17(77.3)	5(22.7)	1		1	
Number of times conceived	Once	81(90.0)	9(10.0)	1			
	≥ Two	78(86.7)	12(13.3)	0.72 (0.28-1.81)	0.48		
Number of ANC check ups	<Four times	9(90.0)	1(10.0)	1.20 (0.14-9.97)	0.86		
	≥Four times	150(88.2)	20(11.8)	1			
Place of delivery	Hospital	155(88.6)	20(11.4)	1			
	Home	4(80.0)	1(20.0)	0.51 (0.05-4.84)	0.56		
Type of delivery	Normal	86(86.9)	13(13.1)	1			
	Cesarean section	73(90.1)	8(9.9)	1.37 (0.54-3.51)	0.500		
Calorie intake among women	Inadequate	157(88.2)	21(11.8)	–			
Protein intake among women	Adequate	7(85.7)	1(12.5)	1			
	Inadequate	150(88.2)	20(11.8)	1.07 (0.12-9.16)	0.95		
Calcium intake among women	Inadequate	157(88.2)	21(11.8)	–			
Iron intake among women	Adequate	2(66.7)	1(33.3)	1			
	Inadequate	155(88.6)	20(11.4)	3.87 (0.33-44.69)	0.27		
Retinol intake among women	Adequate	18(90.0)	2(10.0)	1			
	Inadequate	139(88.0)	19(12.0)	0.81 (0.17-3.78)	0.79		
BMI of mother	Under weight	71(87.7)	10(12.3)	0.82 (0.37-2.209)	0.69		
	Normal weight	69(89.6)	8(10.4)	1			
	Overweight & Obesity	19(86.4)	3(13.6)	0.73 (0.17-3.04)	0.67		
Hemoglobin level of mother	Normal	91(91.0)	9(9.0)				
	Mild anaemia	33(80.5)	8(19.5)				
	Moderate anaemia	14(100.0)	0(0)	–			
	Severe anaemia	19(82.6)	4(17.4)				
**Characteristics of children**							
Age (in months)	Below 24 months	48(82.8)	10(17.2)	1		1	
	25-60 months	111(91.0)	11(9.0)	2.10 (0.83-5.28)	0.11	3.08 (0.86-11.06)	0.08
Sex	Male	76(88.4)	10(11.6)	1			
	Female	83(88.3)	11(11.7)	0.99 (0.39-2.46)	0.98		
Hospital admission in neonatal period	Yes	18(85.7)	3(14.3)	0.76 (0.205-2.85)	0.69		
	No	141(88.7)	18(11.3)	1			
Birth weight	Low birth weight	65(90.3)	7(9.7)	1.39 (0.53-3.65)	0.49		
	Normal weight	93(86.9)	14(13.1)	1			
Immunization status	Fully immunized	155(88.1)	21(11.9)	–			
	Partially immunized	4(100.0)	0(0)	–			
Positive history of Measles in last six months	Yes	8(88.9)	1(11.1)	1.06 (0.12-8.92)	0.95		
	No	151(88.3)	20(11.7)	1			
History of ARI/ADD in last six months	Yes	11(100.0)	0(0)	–			
	No	148(87.6)	21(12.4)	–			
Worm infestation in last six months	Yes	14(93.3)	1(6.7)	1.93(0.24-15.48)	0.53		
	No	145(87.9)	20(12.1)	1			
History of Ingestion of clay/soil	Yes	17(94.4)	1(5.6)	2.39 (0.302-18.98)	0.408		
	No	142(87.7)	20(12.3)	1			
Hospitalization of child in last one year for acute illness	Yes	34(94.4)	2(5.6)	2.58 (0.57-11.64)	0.21	4.68 (0.65-33.68)	0.12
	No	125(86.8)	19(13.2)	1		1	
Calorie intake	Adequate	28(82.4)	6(17.6)	1		1	
	Inadequate	131(89.7)	15(10.3)	1.87 (0.66-5.24)	0.23	0.73 (0.14-3.64)	0.708
Protein intake	Adequate	105(88.2)	14(11.8)	1			
	Inadequate	54(88.5)	7(11.5)	1.02(0.39-2.69)	0.95		
Calcium intake	Adequate	6(85.7)	1(14.3)	1			
	Inadequate	153(88.4)	20(11.6)	1.27(0.14-11.14)	0.82		
Iron intake	Adequate	30(81.1)	7(18.9)	1		1	
	Inadequate	129(90.2)	14(9.8)	2.15 (0.79-5.78)3	0.13	2.82 (0.68-11.55)	0.14
Retinol intake	Adequate	61(85.9)	10(14.1)	1			
	Inadequate	98(89.9)	11(10.1)	1.46 (0.58-3.64)	0.41		
Breast feeding initiation	Immediately after birth	97(90.7)	10(9.3)	1			
	Half an hour-4 hours	58(85.3)	10(14.7)	0.59 (0.23-1.52)	0.28		
	Beyond 4hrs	4(80.0)	1(20.0)	0.41 (0.04-4.05)	0.44		
Pre lacteal feeding to infant	Yes	18(85.7)	3(14.3)	0.76 (0.205-2.85)	0.69		
	No	141(88.7)	18(11.3)	1			
Duration of breast feeding to child (months)	Upto 6 months	8(80.0)	2(20.0)	0.45 (0.08-2.44)	0.35	0.52 (0.07-3.81)	0.52
	7 −12 months	9(75.0)	3(25.0)	0.34 (0.07-1.47)	0.14	0.49 (0.06-4.03)	0.51
	13 −24 months	54(90.0)	6(10.0)	1.02 (0.35-2.97)	0.96	1.62 (0.43-6.09)	0.47
	> 24 months	88(89.8)	10(10.2)	1		1	
Whether mother breastfeeds when she is sick	Yes	119(88.8)	15(11.2)	1			
	No	40(87.0)	6(13.0)	0.84 (0.305-2.31)	0.73		
Whether mother breastfeeds when child is sick	Yes	121(88.3)	16(11.7)	1			
	No	38(88.4)	5(11.6)	1.005 (0.34-2.92)	0.99		
Whether washes hand before feeding the child	Yes	104(88.1)	14(11.9)	1		1	
	No	7(70.0)	3(30.0)	0.31 (0.07-1.35)	0.12	0.47 (0.06-3.45)	0.46
	Sometimes	48(92.3)	4(7.7)	1.61 (0.505-5.16)	0.41	3.71 (0.66-20.79)	0.13
Washes fruits and vegetables	Yes	156(88.6)	20(11.4)	1			
	No	3(75.0)	1(25.0)	0.38 (0.03-3.87)			
Freshly prepares meals	Yes	111(91.0)	11(9.0)	1		1	
	No	48(82.8)	10(17.2)	0.47 (0.18-1.19)	0.11	0.24 (0.07-0.89)	0.03*
Supplementary nutrition utilized by child ICDS	Yes	134(87.6)	19(12.4)	1			
	No	21(91.3)	2(8.7)	–			
	Not sure	4(100.0)	0(0)	–			
Whether house is visited by accredited social health activist (ASHA)/ Community health officer (CHO)^a^	Yes	146(88.5)	19(11.5)	1			
	No	13(86.7)	2(13.3)	0.84 (0.17-4.04)	0.83		
Presence of nearby health facility	Yes	155(88.6)	20(11.4)	1			
	No	4(80.0)	1(20.0)	0.51 (0.05-4.84)	0.56		

Note: Nine children were not tested for anaemia since they were below one year, 10 children refused Hemoglobin test.

(CHO)^a^ – grassroot level workers who are responsible for house visit at the subcenter or primary health center level *statistically significant.

## Discussion

The present study found that malnutrition was highly prevalent among Koraga tribal children, with 81.1% of children being stunted, 78.3% of the children being wasted, and 80.6% of the children being underweight. Malnutrition rates reported in different studies across various states in India range from 8.5% to 75.26% [13,26–32]. According to NFHS-5, stunting, wasting, and underweight are found to be 39.5%, 21.5%, and 35.8% respectively among ST children in Karnataka. However, the overall (non-tribal and tribal children) prevalence of stunting, wasting, and underweight in Udupi district were at 23.1%, 17.6%, and 21.0%, respectively, which is significantly lower than our study findings. The lower rates in Udupi district may be due to the unavailability of ST-specific nutritional data from NFHS-5 [10].

The prevalence rates of malnutrition are higher among children from PVTGs. A study among children belonging to PVTGs of Odisha by Das A et al. found that 75.26% were underweight, 55.42% were stunted, and 60% were wasted [13], while Sunny R et al. also reported a higher percentage of PVTG children (Paniya, Bett Kurumba, Mula Kurumba, and Katunayaka) being malnourished in Nilgiris of Tamil Nadu (63%-underweight; 62%-stunting; 31%-wasting) [26]. Additionally, a study by Narayanappa D et al. on Jenukuruba (PVTG) tribal children in Mysore district found that 62.9% of children under six years of age were malnourished [27]. Similarly, Jaiswal A et al. studied the Irular tribe children in Villupuram, Tamil Nadu, and found that 58.43% were underweight, 64.04% were stunted, and 51.69% were wasted [28]. Although, these studies have documented high levels of malnutrition among PVTG children, the Koraga children in our study exhibited the highest prevalence.

Further, studies among non-PVTG tribal children have also reported higher prevalence of malnutrition; however, the rates are much below the present study findings. A study on Kadukuruba tribal children from Mysore district by Manjunath R et al. reported high malnutrition rates, with 60.4% being underweight, 55.4% with stunting, and 43% with wasting [29]. Similarly, in Wayanad, Kerala, a study by Philip RR et al. conducted among tribal preschool children reported a high prevalence of malnutrition (39%-underweight; 38%-stunting; 20.5%-wasting) [30]. Likewise, another study from southern India, by Reddy VB et al. on Sugali tribal children in Chittoor district, Andhra Pradesh, also reported similar findings (32.7%- underweight;18.3%-wasting; 38.3%-stunting) [31]. However, a study by Duwarah S et al. in a tertiary care hospital in Shillong, Meghalaya, reported a prevalence of underweight at 19.7%, stunting at 35.5%, and wasting at 8.5% among preschool tribal children, which is significantly lower compared to the prevalence observed in our study population [32]. These differences may be attributed to variations in geographical locations and study settings.

In our study, stunting was associated with drinking water from an open well, ≥ two conceptions, low birth weight, poor retinol intake, lack of exclusive breastfeeding, and lack of visits by ASHA, while wasting was linked to older children and inadequate protein intake. A study by Adhikari T et al. found that stunting, wasting, and underweight among tribal children were associated with factors like age, maternal BMI, education, and household wealth. Like the study by Adhikari T et al., which reported a 65.7% prevalence of wasting among children under two years of age, our findings also indicate a similar prevalence (60.3%) in this age group [33].

Our study identified that children aged 25–60 months were having a higher likelihood of wasting, aligning with findings from a study by Meshram II et al. conducted in Integrated Tribal Development Agency (ITDA) areas of nine states across South, East, West, and Central India among tribal children. The study found that children aged 1–3 years were significantly associated with underweight and stunting, while those aged 3–5 years were associated with all three indicators of malnutrition (underweight, stunting, and wasting) [34]. In contrast, a study by Ghosh S et al. among tribal children of Warli, Malhar Koli tribes and Katkari a PVTG of Palghar district, Maharashtra, found no association between age 2–5 years and wasting, while age 3–5 years was linked to underweight, and age 4–5 years had a lower likelihood of stunting [35].

In the present study, low birth weight was a significant predictor of stunting. However, underweight and wasting did not show a significant association. This supports with a study by Singh A et al. among Khasi tribes of Meghalaya, which also found that low birth weight was significantly associated with underweight and stunting [36]. Similarly, a study by Senthilkumar SK et al. in a tribal community of Coimbatore district, which considered malnutrition as the presence of any one of the three parameters consisting of underweight, wasting, or stunting, found that low birth weight babies had a higher risk of malnutrition [37].

Our study did not find any association between the gender of the children, the educational status of parents and indicators of malnutrition. Unlike our findings, study by Sunny R et al. in the tribal population of the Nilgiris, Tamil Nadu, found that male children, age > 2 years, and paternal illiteracy were significantly associated with being underweight, while age > 2 years and maternal illiteracy were associated with stunting and male children and having low socioeconomic status were associated with the wasting [25].

Reducing malnutrition among tribal children needs a comprehensive, multifaceted approach that integrates socio-cultural, environmental, and healthcare considerations. Breaking this vicious cycle requires not only improved nutrition but also enhanced healthcare access, socio-economic support, and targeted health education.

A key strength of this study lies in its comprehensive approach, assessing sociodemographic, maternal, child-related, clinical, and environmental factors. However, the present study also has certain limitations. The study relied on self-reported data for some factors, such as breastfeeding practices and morbidity, which may have led to recall bias. The study used the 24-hour dietary recall method, which may not reflect long-term dietary intake, a detailed dietary analysis is needed for a comprehensive evaluation. The study did not assess specific cultural factors such as dietary practices, food taboos, and traditional cooking methods which could have affected nutritional status of under-five children and should be considered in future research. Few associations observed across stunting, wasting and underweight in the present study were not consistent and showed inverse associations with available biological and epidemiological evidence. These findings may be influenced by the high prevalence of malnutrition and small subgroup sizes in the population studied which could have affected the estimates. Therefore, these associations should be interpreted with caution. Additionally, as the study focused exclusively on under five children from Koraga PVTG community, the findings may not be generalized to other tribal groups.

This study highlights the severe malnutrition burden among Koraga tribal children, with a high prevalence of underweight, stunting, and wasting. Findings of the present study further emphasizes the complex interplay of environmental, dietary, and maternal health factors which contribute to malnutrition. To address the high burden of malnutrition among Koraga tribal children, efforts should focus on strengthening maternal and child healthcare services through improving maternal nutrition, ensuring proper antenatal care, addressing high-risk pregnancies and enhancing ASHA/CHO home visits to prevent low birth weight, which can help reduce malnutrition. Further, continuous growth monitoring, a nutritious diet, better sanitation, access to clean water, and timely healthcare interventions can improve child health outcomes. Policy interventions should ensure targeted nutrition programs under ICDS and Prime Minister’s Overarching Scheme for Holistic Nutrition (POSHAN) Abhiyaan, along with community-led monitoring to improve the effectiveness of existing interventions.

## Supporting information

S1 File Inclusivity in global research. (DOCX)
