# Supplementary material for: Nourishing futures: Assessing nutritional health among children under-five years of age belonging to a particularly vulnerable tribal community in Southern India
Source: PLoS One. 2026 May 11;21(5):e0348880. doi: 10.1371/journal.pone.0348880 (PMC13160329; doi:10.1371/journal.pone.0348880)
Supplement: S1 File — (DOCX) [file pone.0348880.s001.docx]

Inclusivity in global research

PLOS’ policy on inclusivity in global research aims to improve transparency in the reporting of research performed outside of researchers’ own country or community and ensures that PLOS publications reporting global research adhere to high standards for research ethics and authorship. Authors of relevant research articles may be asked to complete the questionnaire below, which outlines ethical, cultural, and scientific considerations specific to inclusivity in global research. This questionnaire may be requested when researchers have travelled to a different country to conduct research, if research uses samples collected in another country, research with Indigenous populations or their lands, or if research is on cultural artefacts. Researchers travelling to another country solely to use laboratory equipment will not normally be required to complete the questionnaire. However, the questionnaire can be requested at the journal’s discretion for any submission – if you have been requested to complete this questionnaire by the PLOS journal you submitted to, please do so.

Please complete the questionnaire below and include this as a Supporting Information file with your manuscript. Note that if your paper is accepted for publication, this checklist will be published with your article in the supporting information files. Please ensure that you reference the checklist in the main body of your manuscript. We suggest adding a subsection ‘Inclusivity in global research’ to your Methods section and adding the following sentence: “Additional information regarding the ethical, cultural, and scientific considerations specific to inclusivity in global research is included in the Supporting Information (SX Checklist)”

The questions have been designed to be applicable to a wide range of study types, and there are subsections for both human subjects research and non-human subjects research. If any of the questions are not relevant to your research please mark them as “N/A” as appropriate.

**Ethical considerations, permits and authorship**

*This section is applicable to all research types.*

Provide details as to who granted permissions and/or consent for the study to take place in the Methods section of your manuscript. This should include the names of **all** ethics boards, governmental organizations, community leaders or other bodies that provided approval for the study. If individuals provided approval refer to these people by their role or title but do not list their name(s).

Ethical approval for the study was obtained from the Institutional Ethics Committee of Kasturba Medical College and Kasturba Hospital, Manipal. Permission from Government was obtained from the Integrated Tribal Development Project (ITDP) Office, Udupi district. Community-level access was facilitated through local tribal community leaders and field health workers prior to data collection.

Reported on page number: 5-6

If there were any deviations from the study protocol after approval was obtained please provide details of these changes in the Methods section of your manuscript.

No deviations from the approved study protocol occurred after ethics approval.

Reported on page number: -

Did this study involve local collaborators that are residents of the country where the research was conducted or members of the community studied? If you do not have any authors from said communities, please provide an explanation for this below.

Yes. The study involved researchers who are residents of Udupi district where the research was conducted. While none of the authors are members of the tribal community studied, tribal community leaders/ representatives and local health workers were involved in facilitating engagement and data collection within the community.

Everyone listed as an author should meet PLOS’ criteria for authorship and all individuals who meet these criteria should be included in the author byline, rather than the acknowledgements. For further information please see the journal’s Authorship Policy.

Authorship was determined in accordance with PLOS authorship criteria.

**Human subjects research (e.g. health research, medical research, cross-cultural psychology)**

Did you obtain written informed consent from a representative of the local community or region before the research took place? How did you establish who speaks for the community? Details of written informed consent obtained from study participants should be reported separately in the Methods section of your manuscript.

Permission from Integrated Tribal Development Project, Udupi District which is an admistritative body by Government of India, and gate keeper permission was obtained from community leaders prior to data collection. Written informed consent was obtained from the mothers of all participating children. For mothers who were illiterate a thumb impression was taken in the presence of a witness along with their signature who could read, write and understand the study related information. Community engagement was facilitated through local leaders and health workers

Reported on page number: 5-6

How did members of the local community provide input on the aims of the research investigation, its methodology, and its anticipated outcome(s)?

When engaging with the local community, how did you ensure that the informed consent documents and other materials could be understood by local stakeholders?

The informed consent forms and participant information sheets were translated into Kannada, the local language. A field investigator proficient in Kannada explained the study objectives, procedures, risks, and benefits verbally in the local language and also shared a copy of participant information sheet. Participants were given the opportunity to ask questions, and written informed consent was obtained only after ensuring adequate understanding.

Prior to data collection, the research team engaged with local health workers and tribal community leaders/ representatives to explain the study objectives and procedures. Discussions were held to ensure that the research questions and methodology were culturally appropriate and feasible within the Koraga community.

Will the findings of the research be made available in an understandable format to stakeholders in the community where the study was conducted (e.g. via a presentation, summary report, copies of publications, etc.)? Please provide details of how this will be achieved.

Yes. Study findings have been shared with Integrated Tribal Development Project office, local health authorities and tribal community leaders/ representatives through summary reports and dissemination meetings. Copies of the published article will also be made available to relevant administrative bodies, including the Integrated Tribal Development Project office.

**Non-human subjects research using specimens/ animals collected as part of the study, or those housed in archival collections. Examples include archaeology, paleontology, botany and zoology.**

Did the permission you obtained from a local authority to perform the study include an agreement on access to outputs and benefit sharing? This may include procedures to enable fair distribution of the benefits and resources arising from the research performed. Please include any details of Prior Informed Consent and Benefit Sharing Agreements obtained. These may be required by field-specific regulations, for example the Convention on Biological Diversity (CBD) and the associated Nagoya Protocol.

Not applicable, as this is a human subject research.

If the material used in your study was imported, please A) provide the year it was imported and B) indicate whether permits were obtained to import/export the materials used, C) provide details of any permits obtained. If this information is not available, please indicate this.

Not applicable, as this is a human subject research.

If you used archival specimens, please state how the material used in your study was acquired by the institute it is held in and provide details of any permits obtained for the original excavations/ sample collection. If this information is not available, please indicate this.

Not applicable, as this is a human subject research.

How was the potential cultural significance of the materials collected in your study to local communities considered in your research design? Were Indigenous peoples and/or local researchers and institutions involved with archaeological excavations / collection of specimens? If so, please provide a description of their involvement.

Not applicable, as this is a human subject research.

If your manuscript includes photographs of human remains please indicate whether authors obtained permission from descendants or affiliated cultural communities to do so.

Not applicable, as this is a human subject research.
