# Supplementary material for: Nourishing futures: Assessing nutritional health among children under-five years of age belonging to a particularly vulnerable tribal community in Southern India
Source: PLoS One. 2026 May 11;21(5):e0348880. doi: 10.1371/journal.pone.0348880 (PMC13160329; doi:10.1371/journal.pone.0348880)
Supplement: Attachment — Submitted filename: Responses to reviewers.docx [file pone.0348880.s003.docx]

**Journal Requirements:**

When submitting your revision, we need you to address these additional requirements.

1. **Please ensure that your manuscript meets PLOS ONE's style requirements, including those for file naming. The PLOS ONE style templates can be found at**

[**https://journals.plos.org/plosone/s/file?id=wjVg/PLOSOne_formatting_sample_main_body.pdf**](https://journals.plos.org/plosone/s/file?id=wjVg/PLOSOne_formatting_sample_main_body.pdf) **and**

[**https://journals.plos.org/plosone/s/file?id=ba62/PLOSOne_formatting_sample_title_authors_affiliations.pdf**](https://journals.plos.org/plosone/s/file?id=ba62/PLOSOne_formatting_sample_title_authors_affiliations.pdf)

The Manuscript has been formatted according to Plos One style requirement including file naming

**2. Please include a complete copy of PLOS’ questionnaire on inclusivity in global research in your revised manuscript. Our policy for research in this area aims to improve transparency in the reporting of research performed outside of researchers’ own country or community. The policy applies to researchers who have travelled to a different country to conduct research, research with Indigenous populations or their lands, and research on cultural artefacts. The questionnaire can also be requested at the journal’s discretion for any other submissions, even if these conditions are not met.  Please find more information on the policy and a link to download a blank copy of the questionnaire here:** [**https://journals.plos.org/plosone/s/best-practices-in-research-reporting**](https://journals.plos.org/plosone/s/best-practices-in-research-reporting)**. Please upload a completed version of your questionnaire as Supporting Information when you resubmit your manuscript.**

Thank you for the guidance. We have completed PLOS’ questionnaire on inclusivity in global research and uploaded it as Supporting Information (S1 Checklist) along with the revised manuscript. A corresponding statement referencing the checklist has also been added to the Methods section of the manuscript.

3. **We note that your Data Availability Statement is currently as follows: All relevant data are within the manuscript and its Supporting Information files.

Please confirm at this time whether or not your submission contains all raw data required to replicate the results of your study. Authors must share the “minimal data set” for their submission. PLOS defines the minimal data set to consist of the data required to replicate all study findings reported in the article, as well as related metadata and methods (**[**https://journals.plos.org/plosone/s/data-availability#loc-minimal-data-set-definition**](https://journals.plos.org/plosone/s/data-availability#loc-minimal-data-set-definition)**).

For example, authors should submit the following data:

- The values behind the means, standard deviations and other measures reported;
- The values used to build graphs;
- The points extracted from images for analysis.

Authors do not need to submit their entire data set if only a portion of the data was used in the reported study.

If your submission does not contain these data, please either upload them as Supporting Information files or deposit them to a stable, public repository and provide us with the relevant URLs, DOIs, or accession numbers. For a list of recommended repositories, please see** [**https://journals.plos.org/plosone/s/recommended-repositories**](https://journals.plos.org/plosone/s/recommended-repositories)**.

If there are ethical or legal restrictions on sharing a de-identified data set, please explain them in detail (e.g., data contain potentially sensitive information, data are owned by a third-party organization, etc.) and who has imposed them (e.g., an ethics committee). Please also provide contact information for a data access committee, ethics committee, or other institutional body to which data requests may be sent. If data are owned by a third party, please indicate how others may request data access.**

The dataset contains information collected from members of a Particularly Vulnerable Tribal Group (PVTG), which may include potentially sensitive community-level data. Therefore, the de-identified dataset cannot be made publicly available to protect participant confidentiality and community privacy, in accordance with the conditions of approval granted by the Institutional Ethics Committee of Kasturba Medical College and Kasturba Hospital, Manipal. However, the minimal dataset required to replicate the findings of this study may be made available upon reasonable request to the corresponding author, subject to review and approval by the Institutional Ethics Committee.

4. **If the reviewer comments include a recommendation to cite specific previously published works, please review and evaluate these publications to determine whether they are relevant and should be cited. There is no requirement to cite these works unless the editor has indicated otherwise.**

Thank you for the clarification. No specific previously published works were recommended for citation in the reviewer comments. We have carefully reviewed the manuscript and ensured that relevant literature has been appropriately cited.

5. **Please review your reference list to ensure that it is complete and correct. If you have cited papers that have been retracted, please include the rationale for doing so in the manuscript text, or remove these references and replace them with relevant current references. Any changes to the reference list should be mentioned in the rebuttal letter that accompanies your revised manuscript. If you need to cite a retracted article, indicate the article’s retracted status in the References list and also include a citation and full reference for the retraction notice.**

Thank you for this suggestion. We have carefully reviewed the entire reference list to ensure completeness and accuracy.

The reference no 5 by Singh DN (2022) has been replaced for better reference with a more relevant and reliable study: Kshatri JS et al., 2025.

The web reference number 14 on Particularly Vulnerable Tribal Groups from Vikaspedia has also been replaced with a peer-reviewed article: Sabar B., 2016

Additionally, the following relevant references have been added to strengthen the manuscript and provide appropriate background and methodological support.

Reference no. 18 Soman B et al., 2025, which provides ethnographic insights into healthcare access among a Particularly Vulnerable Tribal Group in South India.

Reference no. 23 World Health Organization, 2006 Child Growth Standards, used to define anthropometric indicators of malnutrition.

Furthermore, duplicate references identified in the list have been removed. All in-text citations, numbering, and reference formatting have been updated accordingly throughout the manuscript.

**Response to reviewers' comments:**

**Please use the space provided to explain your answers to the questions above. You may also include additional comments for the author, including concerns about dual publication, research ethics, or publication ethics. (Please upload your review as an attachment if it exceeds 20,000 characters)**

**Reviewer #1:**

**The article gives information about the nutritional status of PVTG's children below 5 years of age. Most of the terms mentioned in the article are technical terms used by medical practitioners which may not be understood by the researchers of other fields. Explaining those terms in the footnotes would have made the readers understand the article better.**

Thank you for this valuable suggestion. We agree that certain technical and clinical terms may not be familiar to readers from non-medical disciplines. Accordingly, we have revised the manuscript to define key terms at their first occurrence within the text to improve clarity and accessibility.

Line number: 139-144

**Even though the statistical analysis was rigorous the mention of cultural factors was missing in the article which might also play a significant role in the determining the reasons for the stunned growth.**

Thank you for this valuable observation. We agree that cultural factors may play an important role in influencing nutritional outcomes among tribal children. In the present study, certain cultural aspects such as breastfeeding practices, colostrum feeding, weaning practices, history of illness, hospitalization, parasitic infestation, and health-seeking behaviour were assessed. However, broader cultural factors such as dietary practices, food taboos, and traditional cooking practices were not specifically explored within the scope of this study. This has been acknowledged as a limitation in the revised manuscript.

Line number: 333-335

**Some of the associations concerning the malnutrition was ambiguous.**

Thank you for this observation. The associations reported in the manuscript are based on the statistical analysis performed. Some findings showing inverse associations may reflect the high prevalence of malnutrition in the study population and small sample size in the categories analysed. We have clarified this point and acknowledged it as a limitation of the study.

Line number: 335-340

**Even though the article mentions about ITDS, ASHA etc their role concerning the malnutrition was not clear.**

Thank you for highlighting the point. We have revised the manuscript to clearly describe the role of Integrated Child Development Services (ICDS), Integrated Tribal Development Project (ITDP) and Accredited Social Health Activists (ASHAs) in addressing malnutrition.

Line number: 114- 116 and 126-131

**Reviewer #2: 1. Yes, it is a technically sound piece of scientific research with data that supports the conclusions.
2. Yes, the analysis has been performed appropriately.
3. Yes, it is.
4. The manuscript is written in a standard english.**

The study addresses an important public health issue, as nutritional health among under five years children in Particularly Vulnerable Tribal Groups (PVTGs) remains a serious concern. The data appear to support the conclusions, and the statistical analyses have been conducted appropriately. However, several revisions are required to strengthen the scientific rigor and clarity of the manuscript.

**The Introduction should include a brief description of the Koraga tribal community to provide adequate contextual background.**

Thank you for this suggestion. A brief description of the Koraga tribal community has now been added to the introduction to provide contextual background and help readers understand the sociocultural setting of the study population.

Line number: 84-91

**The rationale for limiting the survey to 180 participants needs to be clearly explained. It should be specified whether this number represents the total population of under five children belonging to the Koraga community in the selected district or whether other factors determined the sample size.**

Thank you for this important clarification. The initial sample size was calculated to be 291. However, all Koraga tribal hamlets in the selected study area were visited, and all eligible under-five children available during the study period were included. Therefore, the final sample of 180 represents the total accessible under-five Koraga population in the study setting. This explanation has now been clarified in the Methodology section

Line number: 111 – 113

**In the Methodology section, the sampling procedure must be described in detail. The manuscript should clearly explain how the sample of 180 children was identified and recruited, including the underlying sampling framework and any inclusion or exclusion criteria.**

Thank you for the valuable comment. The sampling procedure has been revised in the methodology section to provide greater clarity. A complete enumeration sampling approach was adopted. All tribal hamlets in study setting were visited and all eligible children under five years of age were included. The final sample of 180 children represents all accessible and eligible under-five children in the study setting. The revised description has been made clearer in the manuscript.

Line number: 111-122

**For infants and young children from birth to 24 months, it is essential to mention that, as per WHO recommendations, nutritional status was assessed using weight-for-length, along with length-for-age and weight-for-age indicators.**

Thank you for this important correction. Nutritional status assessment for infants and young children aged below 24 months was conducted using WHO-recommended anthropometric indicators. This clarification has been revised in the methodology section.

Line number: 139-141

**In the Results section, Table 2 reports data for 178 participants instead of 180. A clear explanation should be provided for this discrepancy, including whether any participants were excluded from the final analysis and the reasons for such exclusion. This information should be explicitly stated in the manuscript.**

Thank you for the observation. Table 2 presents characteristics of the surveyed mothers. A total of 178 mothers participated in the study. Although 180 children under five years of age were included overall, one mother had twins and another mother had two children under five years. Hence, maternal characteristics in Table 2 reflect 178 mothers. This clarification has now been added to the table footnote.

Line number 192– 193

**Finally, as the study focuses on under five years PVTG children, a dedicated section on study limitations is required.**

Thank you for this suggestion. A statement on study limitations has already been included in the manuscript, and we have further clarified that the findings are specific to under-five children from the Koraga PVTG community and may not be generalizable to other tribal populations.

Line number: 331-341
